# Regarding Emitter Positioning for Nanoflow Electrospray Ionization with a High-Capacity Inlet Capillary

**DOI:** 10.1021/jasms.5c00441

**Published:** 2026-02-06

**Authors:** Noah M. Lancaster, Scott T. Quarmby, Katherine A. Overmyer, Joshua J. Coon

**Affiliations:** † Department of Chemistry, 5228 University of Wisconsin-Madison, Madison, Wisconsin 53706, United States; ‡ Department of Biomolecular Chemistry, University of Wisconsin–Madison, Madison, Wisconsin 53706, United States; § 145254 Morgridge Institute for Research, Madison, Wisconsin 53715, United States

**Keywords:** nanoflow electrospray ionization, proteomics, inlet capillary, emitter positioning, ion plume characterization

## Abstract

Nanoflow electrospray ionization is commonly used for proteomics due to its high sensitivity. Signal intensity, however, is dependent on optimal emitter positioning relative to the mass spectrometer inlet. Here, we characterize the effect of varied emitter positions on peptide signal intensity in all three dimensions using emitters and flows consistent with standard proteomic analyses. We observe improved signal robustness to *x*/*y* variations at increasing *z* distances and demonstrate that positioning within 1 to 2 mm of the optimal location will maintain consistent signal. Signal intensity behavior is consistent across the *m*/*z* range, suggesting emitter positions do not need to be fine-tuned for different analytes for proteomics analyses. These results provide insight for proteomics researchers using nanoflow LC–MS/MS.

## Graphical abstract

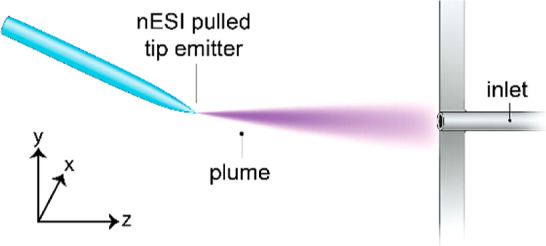

## Introduction

Owing to its high sensitivity, nanoflow electrospray liquid chromatography coupled with tandem mass spectrometry (nESI-LC-MS/MS) is widely used for protein sequence analysis.^1,2^ Whereas high-flow electrospray sources typically have relatively fixed emitter alignments and are more robust to changes in emitter positioning, nESI sources frequently require manual alignment in three dimensions.^3,4^ Since the number of ions reaching the MS system can vary depending on the location of the nESI emitter, positioning is a critical aspect of achieving robust and reproducible results.

Multiple reports illustrate the relationship between emitter positioning and electrospray signal intensity;^2,4–10^ however, these studies typically examine a single dimension or evaluate emitters and flow regimes not typical of shotgun proteomic workflows. To understand these relationships, we investigate here the effect of emitter positioning on signal intensity when using fused-silica capillaries with integrated emitters at a flow rate of 300 nL/min, a common choice across the field.^11–17^ Experiments were conducted using an Orbitrap Ascend mass spectrometer, a notable example of instruments commonly used in proteome analysis,^18^ which features a nanoflow source and asymmetric inlet capillary (high-capacity transfer tube, HCTT). This work represents, to our knowledge, the first report in the literature characterizing how emitter positioning impacts signal intensity for nanoflow electrospray ionization into an inlet capillary without radial symmetry.

Since most nESI emitters are manually positioned, understanding the precision in positioning that is required could assist in reducing batch-to-batch variation that is common in larger proteomic experiments. Further, recent efforts to improve the throughput of proteome analysis feature the use of multiple columns with multiple emitters aligned with the source at the same time.^19–23^ By performing measurements in all three dimensions, this report provides insight for implementing such a multiemitter setup.

## Experimental Section

Emitter positioning measurements were performed by infusing BSA peptides at 300 nL/min into an Orbitrap Ascend mass spectrometer (Thermo Scientific) using a fused silica capillary (360 μm O.D, 75 μm I.D.) with integrated emitter (∼10 μm opening).^24^ The emitter was aligned to the inlet using the source camera and the *x*/*y*/*z* micrometer on a NanoSpray Flex source (Thermo Scientific). The origin of the coordinate system used here was defined as the center of the inlet capillary opening. In particular, the *z* = 0 plane is orthogonal to the opening of the inlet capillary, and the *x* = 0 and *y* = 0 planes are parallel to the inlet capillary axis. Top- and side-view images of the starting position are shown in Figure S2. Code used for data analysis is available at https://github.com/coongroup/EmitterPositioning. Raw data is available at MassIVE accession number MSV000100165. Additional experimental and data analysis details are described in the Supporting Information.

## Results and Discussion

We characterized signal intensity dependence on emitter position for nanoflow electrospray by infusing a mixture of peptides generated via trypsin digestion of purified BSA in an Orbitrap-quadrupole linear ion trap hybrid mass spectrometer (Orbitrap Ascend Tribrid). To ensure LC–MS applicability, we used an emitter size (∼10 μm orifice) and flow rate (300 nL/min) identical to those typical of capillary LC–MS.^11–17^ The resulting MS1 analysis produced numerous ions across the *m*/*z* range (Figure S3). We selected two of these ions, a doubly protonated species (LGEYGFQNALIVR at *m*/*z* 740.4) and a triply protonated species (RHPEYAVSVLLR at *m*/*z* 480.6), for their strong signal intensity and stability over the time range of the experiments here (Figures S4 and S5). With the above setup, we tracked those *m*/*z* peaks as a function of emitter position in three dimensions and recorded intensity distributions (Figure 1). Figure 1C presents the ion signal across x-positions, where the *x* = 0 plane is aligned to the central axis of the inlet capillary and the dashed lines depict the MS inlet opening width in this dimension (0.6 mm, Figure S6).^25,26^ Strikingly, we observe that the signal is relatively stable and high across two millimeters of the *x*-dimension. Further, greater than 50% of the ion signal is retained at distances up to ∼five times the width of the inlet (i.e., full width half max (fwhm)). Figure 1D presents the same concept but in the y-dimension, where good signal stability is also observed across a similar length of two millimeters; however, the larger inlet opening in the y-dimension (1.6 mm) does not appear to proportionally impact the width of the intensity distribution. The ion plume is roughly symmetrical in both *x*- and *y*-dimensions;^8^ therefore, it is expected that the smaller inlet width in the *x*-dimension would have a higher probability of being in the high density region of the plume than in the *y*-dimension. Proportional to inlet opening, the *x* position of the emitter would consequently have less of an effect on the intensity than the *y* position. We note the intensity distributions appear to be slightly off-center; however, we find the centroids of the distributions (−0.1 and 0.3 mm, *x*- and *y*-respectively, Figure S7) were within the tolerance of the micrometer. Figure 1E presents the ion intensities as a function of emitter position in the *z*-dimension. Impressively, signal continues to be observed up to ∼7 mm removed from the inlet at levels close to 50% of the highest. Not surprisingly, the highest signal is observed at the closest position, but, as noted above, that signal gradually declines with increasing distance. The relative intensities of these two peptide ions remain fairly constant at all observed z positions (Figure S8). Overall, identical trends were observed for both peptides over all dimensions. As these peptides are fairly high signal intensity, we wanted to confirm that the trends reported here were not biased by signal saturation, so we extracted signal intensities for low intensity features and compared the *x*, *y*, and *z* intensity distributions (Figure S9). This analysis confirms that these two peptides are representative of global trends for our tryptic peptide sample.

**Figure 1:**
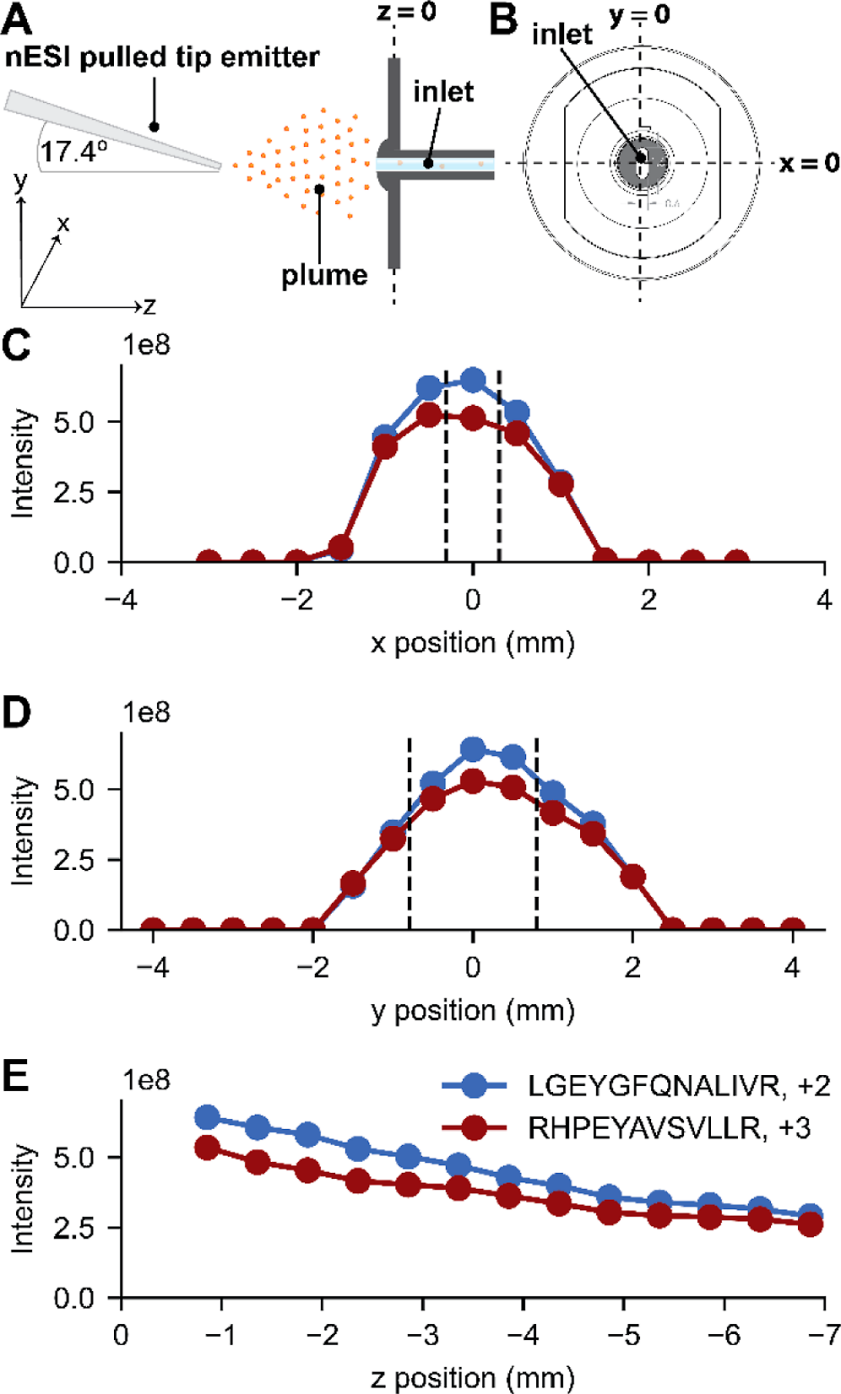
1D Positioning Experiments. (A) The *x*/*y*/*z* coordinate system used for this study. (B) An *x*/*y* perspective of the inlet with coordinate definitions. (C) Intensity dependence on *x*-position for two selected peptides (at *y* = 0 and *z* = −0.9). Dotted lines indicate the estimated position of the inlet capillary edges. (D) Intensity dependence on *y*-position for two selected peptides (at *x* = 0 and *z* = −0.9). Dotted lines indicate the estimated position of the inlet capillary edges. (E) Intensity dependence on *z*-position for two selected peptides (at *x* = 0 and *y* = 0).

With these unidimensional observations complete, we next characterized the three-dimensional interplay of emitter position. For these experiments, we rastered the emitter across *x*, *y*, and *z* dimensions (Figures 2 and S10). A key observation from these data is that signal intensity becomes more robust to *x*- and *y*-positioning as distance from the inlet increases (*z*-position). This observation is likely explained by a widening and flattening of the ion plume spatial distribution with increasing distance as previously reported.^9^ Specifically, with the emitter close to the inlet (*z* = −0.9 mm), the *x*, *y* tolerance to retain ∼90% signal is ∼1 mm, whereas at *z* = −4.9 mm the tolerance is ∼2–3 mm (Figure S11). To our knowledge, these data are the first to map the three-dimensional space of the nESI plume in the context of the conditions that are typical in shotgun proteomics (i.e., capillary LC–MS).^2,6,8–10,27–35^ An especially exciting potential application of this knowledge is in the use of multiple emitters and/or parallel separations. In these cases, two or more emitters must be simultaneously positioned in front of the inlet, inhibiting the conventional alignment approaches where you put the emitter as centered and close as possible. Here, one desires to both achieve maximal ion signal while ensuring similar performance across the emitters. In such scenarios, our data suggest that moving 3–5 mm back in the *z*-dimension will ease the constraints on the *x*,*y*-positioning and reduce differences between the multiple emitters.

**Figure 2:**
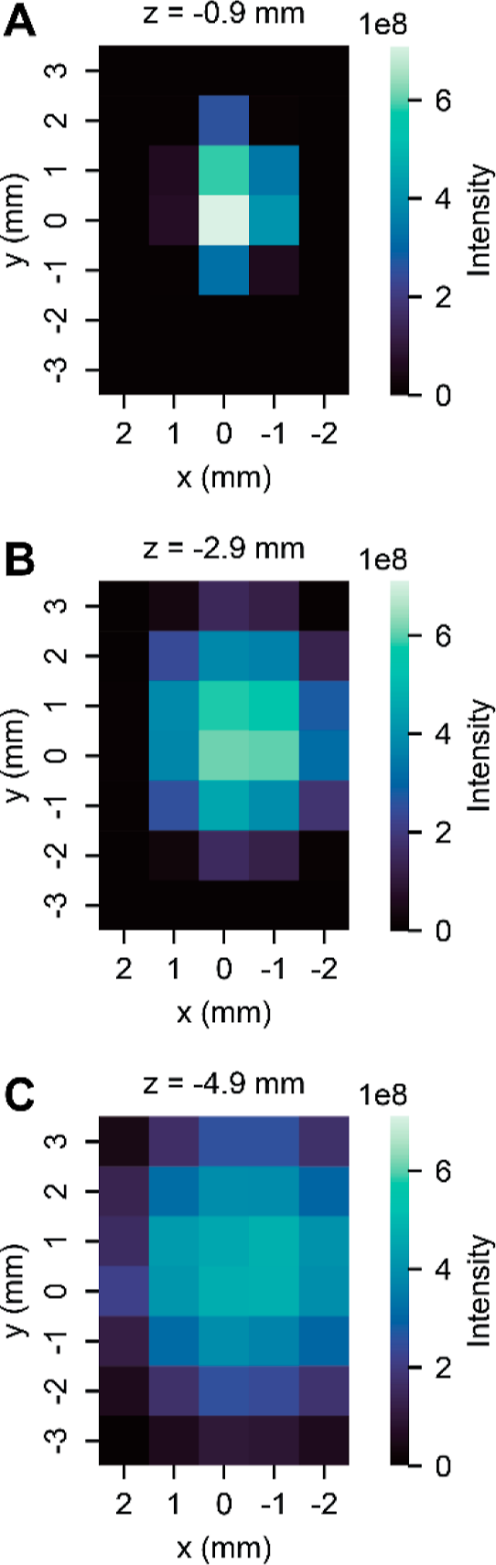
2D Positioning Experiments. Heatmaps showing intensity of LGEYGFQNALIVR (+2) at different x/y positions at (A) *z* = −0.9 mm, (B) *z* = −2.9 mm, and (C) *z* = −4.9 mm.

The source used in this study presented the emitter such that the central axis of the emitter is at an angle of ∼17.4° relative to the *y*-axis (Figures 1A and S2B). Other nanoflow sources feature angles ranging from 0° to 90°.^4,36–38^ From a geometric perspective, this angle could shift the *y*-dimension ion intensity distribution to increasingly positive values as the emitter is positioned further away in the *z*-dimension (see Supplementary Note, Figure S1, Table S1) due to the contributions of the initial momentum of the charged particles as they leave the emitter. However, we find the centers of the *y*-dimension intensity distributions are consistent at different *z*-positions suggesting the angle had little to no impact (Figure S12). Gas flow into the inlet capillary has a collimating effect on the electrospray plume and is the dominating factor controlling ion trajectories due to its influence on the aerodynamic flow of the charged particles.^28,39^ Additionally, the electrical potential difference between the emitter and the inlet capillary could curve the ion plume toward the inlet. These two effects would dominate especially as desolvation decreases droplet size over time, increasing the mobility. These mechanisms likely explain why the geometric effect of the angled emitter axis relative to the *y*-axis is not observed.

Finally, to ensure there are no broader analyte specific effects at play, we looked at the effect of position across various *m*/*z* peaks (*n* = 243, charge >1, *m*/*z* values ranging from ∼350 to ∼1250 *m*/*z*) stemming from the BSA tryptic digest. We plotted full-width half-maximum (fwhm) values for the *x* and *y* intensity distributions at the closest *z* position (Figure 3A,B) and the intensity fold changes between the closest and furthest positions (i.e., *z* = −0.9 and *z* = −6.9, Figure 3C) vs *m*/*z*. Although there is variation in measured fwhm values and fold-change across analytes, this variation is not correlated with *m*/*z*. Note that outliers in these plots correspond to lower intensity peaks more likely to exhibit unstable signal for our setup (Figure S13). We conclude that the effect of emitter positioning on signal intensity does not strongly depend on the analyte for peptide analyses, such that the same emitter position can be appropriate for most peptides.

**Figure 3:**
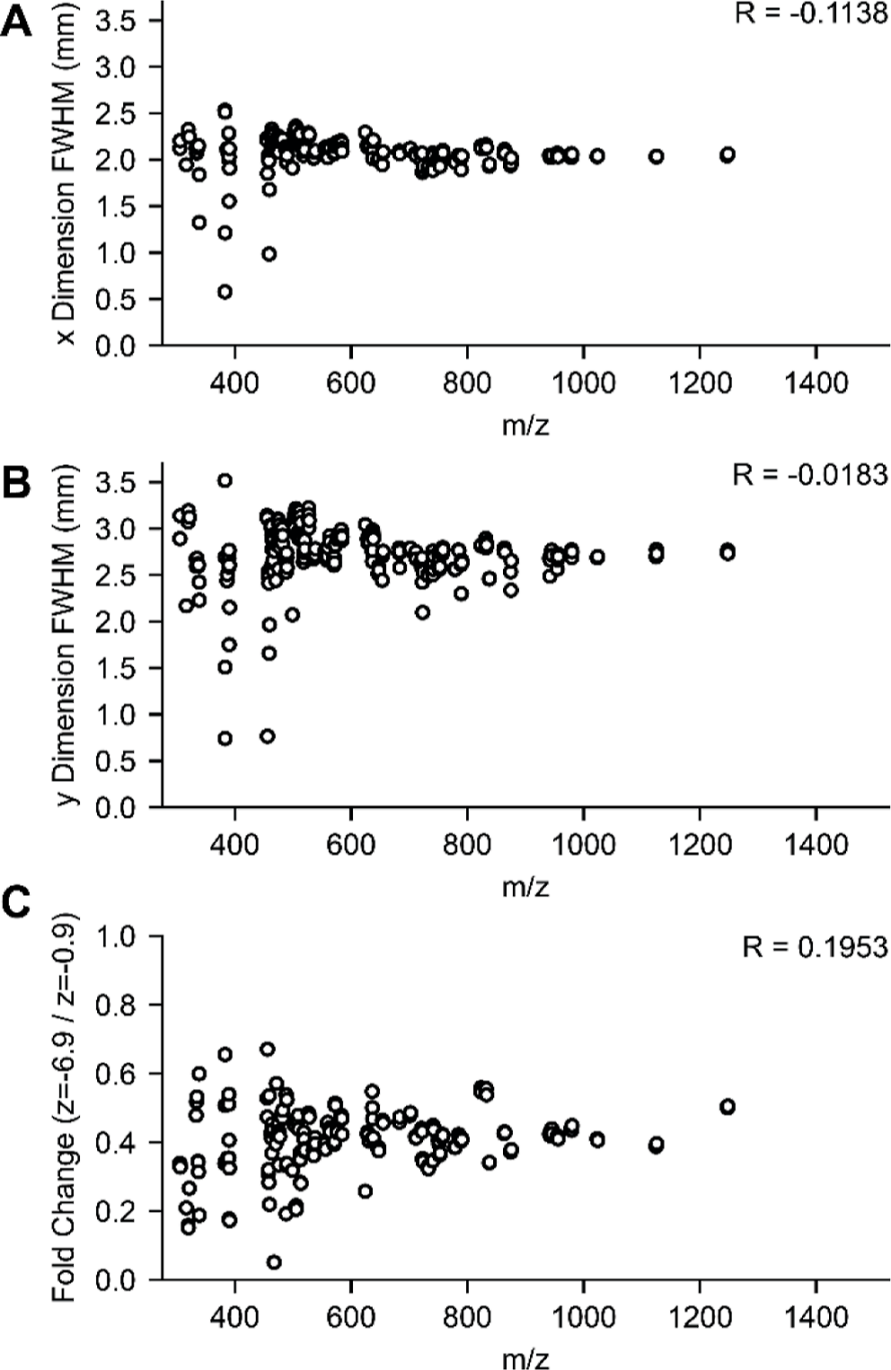
Dependence of Emitter Position Results on *m*/*z*. Mass spectra for the two extreme z-positions (at *x* = 0 and *y* = 0) were filtered for *m*/*z* peaks present in both with S/N > 300 and charge >1. The full-width half-maximum (fwhm) was approximated via spline interpolation for the (A) *x*-distribution and (B) *y*-distribution at *z* = −0.9 mm, and (C) the fold change between the extreme *z*-positions was calculated (at *x* = 0, *y* = 0 mm). The Pearson correlation, R, of these values with *m*/*z* was calculated and is included at the top right of each panel.

## Conclusions

We report the dependence of peptide signal intensity on emitter positioning for nanoflow electrospray. Specifically, within 1–2 mm in any dimension, one can achieve reasonably consistent and robust signal. Distinct intensity profiles we observed here for the *x*- and *y*-dimensions likely arise from the asymmetric shape of the ion capillary. We demonstrate improved robustness of signal intensity to *x*/*y* variation at increasing *z* distances, an observation that will be helpful for positioning multiple emitters, for example.^19–23^

We provide evidence that the effect of the emitter position on signal intensity does not strongly depend on the analyte for bottom-up proteomics analysis. This report provides insight into the role of emitter positioning on signal intensity for bottom-up proteomics and represents the first characterization of the effect of emitter position on electrospray signal intensity on an instrument with an inlet capillary lacking radial symmetry. Future areas of interest would be an examination of how trends vary across different flow rates or emitter types, as well as assessing how the exact intensity profiles vary across instruments with differences in their atmospheric interface.

## Supplementary Material





## Notes

Supplementary results. Supplementary Methods, Supplementary Note, Figure S1 (Geometric Diagram of Nano-Electrospray Emitter Configuration), Table S1 (Predicted Offsets (Δ*y*) at Different z Distances), Figure S2 (Emitter Position Images Recorded with Source Camera), Figure S3 (MS1 Spectra of Infused BSA Tryptic Digest), Figure S4 (Signal Stability of Infused Peptides), Figure S5 (QC Measurements for Emitter Positioning Experiments), Figure S6 (Inlet Capillary Drawing), Figure S7 (Estimating Distribution Centroids), Figure S8 (Dependence of Precursor Intensity Ratio on z Position), Figure S9 (Dependence of Signal Intensity on Emitter Position for Low Level Precursor Ions), Figure S10 (2D Positioning Experiments for RHPEYAVSVLLR), Figure S11 (Width at 90% Maximum as Function of z Position), Figure S12 (Dependence of y Intensity Distribution on z Position), Figure S13 (Dependence of Emitter Position Results on Peak Intensity) (PDF) (PDF)
Supplementary Table 1: Data Description and Processing Results (XSLX) (XLSX)
