# Supplementary material for: Regarding Emitter Positioning for Nanoflow Electrospray Ionization with a High-Capacity Inlet Capillary
Source: J Am Soc Mass Spectrom. 2026 Feb 6;37(3):754–9. doi: 10.1021/jasms.5c00441 (PMC12893671; doi:10.1021/jasms.5c00441)
Supplement: Supplementary file 1 [file js5c00441_si_001.pdf]

Supporting Information

Regarding Emitter Positioning for Nanoflow Electrospray Ionization with a High-Capacity Inlet Capillary

Noah M. Lancaster[1,2], Scott T. Quarmby[2], Katherine A. Overmyer[2,3], and Joshua J. Coon[1,2,3 *]

[1] Department of Chemistry, University of Wisconsin-Madison, Madison, WI 53706, USA
[2] Department of Biomolecular Chemistry, University of Wisconsin-Madison, Madison WI 53706, USA
[3] Morgridge Institute for Research, Madison, WI 53715, USA
* Correspondence: J.J.C. (coon@wisc.edu)

Supplementary Methods

Materials and Reagents

Ultrapure water was generated with a Barnstead GenPure Pro system (Thermo Scientific). Tris(2-carboxyethyl)phosphine hydrochloride (TCEP, C4706-2G), chloroacetamide (≥98%, C0267-100G), urea (U5378-1kg), and trifluoroacetic acid (TFA, HPLC grade, >99.9%) were obtained from Sigma-Aldrich. Tris Buffer (1 M Tris pH 8.0, 0.2 μm filtered, Invitrogen, AM9856), formic acid (LC-MS grade), acetonitrile (Optima LC/MS grade), and bovine serum albumin (BSA, Thermo Fisher Scientific, 23209, Albumin Standard) were obtained from Fisher Scientific. Trypsin (Sequencing Grade Modified Trypsin, V5113) was purchased from Promega. LysC (Lysyl Endopeptidase, 100369-826) was acquired from VWR.

Sample Preparation

BSA standard was diluted to 0.5 mg/mL with a final composition of 2 M Urea/100 mM Tris/2.5 mM TCEP/10 mM chloroacetamide. LysC was added at a 1:50 enzyme:BSA ratio and the sample was rocked at ambient conditions for 4 hours. Trypsin was added at a 1:50 enzyme:BSA ratio and the sample was rocked at ambient conditions overnight. Digestion was quenched with 10% TFA added to pH <2. The sample was centrifuged for 5 minutes at 9,000 g, desalted using a Strata-X 33 μm polymeric reversed phase SPE cartridge (Phenomenex), dried in a SpeedVac (Thermo Scientific), and resuspended in 0.2% formic acid. Peptide concentration was measured via NanoDrop (Thermo Scientific), and the sample was diluted to 0.5 mg/mL with a final solvent composition of 24% acetonitrile/0.2% formic acid.

Data Collection

Emitter positioning measurements were performed by infusing BSA peptides into an Orbitrap Ascend mass spectrometer (Thermo Scientific) with a Vanquish Neo UHPLC (Thermo Scientific). An emitter was prepared by laser pulling and etching a fused silica capillary (360 μm OD, 75 μm ID) with ~10 μm tip ID[1], connecting to the nanoLC, and aligned to the inlet with a NanoSpray Flex source (Thermo Scientific) and the source camera. The x = 0 and y = 0 planes were defined as parallel to the central axis of the inlet capillary in these dimensions, and z = 0 was defined at the plane orthogonal to the inlet opening. Note that the xyz stage positions were indicated in intervals of 1 mm, so the uncertainty of the xyz positioning is ±0.5mm. Positioning results are reported accordingly. **Figure S2** shows top and side-view images of the starting position**.** The emitter angle in the y-dimension was measured to be ~17.4° using the ImageJ software.[2] Infusion was performed by injecting 20-22 μL using Direct Control within Xcalibur at a flow rate of 300 nL/min and mobile phase conditions identical to the sample solvent composition. The infusion method provided over an hour of stable signal (**Figure S4**). For each set of positioning experiments, an infusion was begun, and the signal was monitored until it had stabilized (~15 min). Then, the emitter position was varied for data collection for no more

than approximately 30 minutes to ensure stable spray. For this study, this procedure was performed once to collect data for the 1D experiments shown in **Figure 1** and was performed once at each z position to generate the data in **Figure 2**. Measurements were taken at the starting positions throughout each data collection stage to confirm consistent signal intensity (**Figure S5**).

A spray voltage of 2000 V was applied with transfer tube temperature of 275°C. Orbitrap MS1 scans were acquired at 60,000 resolving power with scan range 300–1500 *m/z*, 30% RF Lens setting, 250% Normalized AGC Target, and maximum injection time of 100 ms. Signal intensity was allowed to stabilize after adjusting positions. For each position, 100 scans were acquired in the Tune software.

Data Analysis

An *in silico* BSA digest was performed using PeptideMass in Expasy[3] with two missed cleavages allowed. Outputs were used to select a doubly and triply charged precursor ($LGEYGFQNALIVR^{2+}$ and $RHPEYAVSVLLR^{3+}$) with high signal intensity and stability. Ion intensity was averaged across scans with a 15-ppm *m/z* tolerance using the 'pymsfilereader' library in Python. Calculation of approximate full-width half-maximum (FWHM) values for intensity distributions was performed by fitting the data with the 'UnivariateSpline' function within SciPy.[4] For the global comparisons across *m/z*, mass spectra for the two extreme z-positions (at x = 0 and y = 0) were averaged in Freestyle, followed by exporting peak lists and filtering peaks present in both spectra (within 5 ppm) for charge > 1 and S/N>300. This filtering retained higher intensity peaks more likely to be reliable indicators for the effect of emitter positioning on signal intensity. Pearson correlations were calculated using 'pearsonr' within SciPy.[4]

Supplementary Note

The Nanospray source used in this study exhibits a ~17.4° angle between the central axis of the emitter and the central axis of the inlet capillary in the y dimension. An illustration of this geometry is shown below, where θ represents the angle, d_z represents the distance of the emitter from the inlet in the z dimension, and Δy represents the offset between the emitter's y position and the y position at the intersection of the central axis of the emitter and the plane of the inlet capillary.

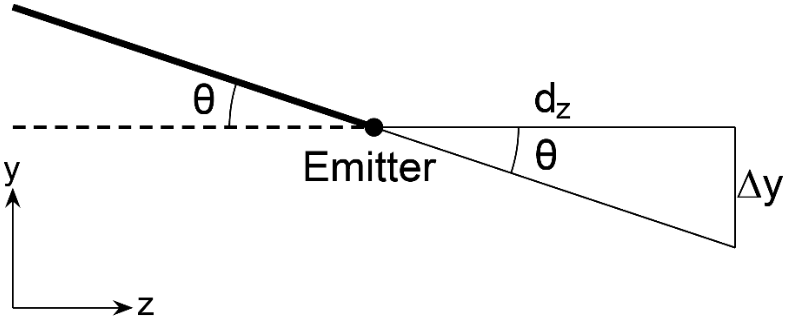

Figure S1: Geometric Diagram of Nano-Electrospray Emitter Configuration.

For an angle, θ, of 17.4° and an emitter distance, d_z, the offset, Δy, is defined by

$$d_z \tan\theta$$

Thus, for the z positions shown in **Figure 2** and **Figure S10**, the offsets are calculated as

Table S1: Predicted Offsets (Δy) at Different z Distances.

Absolute z Distance (mm)	Theoretical Δy (mm)
0.9	0.3
2.9	0.9
4.9	1.5

This feature of the source geometry would be expected to impact the emitter positioning results in the y dimension if the geometric configuration of the source was a major driver of ion trajectories. However, this trend is not clearly observed as shown in **Figure S12**.

References

(1) Shishkova, E.; Hebert, A. S.; Westphall, M. S.; Coon, J. J. Ultra-High Pressure (>30,000 Psi) Packing of Capillary Columns Enhancing Depth of Shotgun Proteomic Analyses. *Anal. Chem.* **2018**, *90* (19), 11503–11508. https://doi.org/10.1021/acs.analchem.8b02766.

(2) Schindelin, J.; Rueden, C. T.; Hiner, M. C.; Eliceiri, K. W. The ImageJ Ecosystem: An Open Platform for Biomedical Image Analysis. *Molecular Reproduction and Development* **2015**, *82* (7–8), 518–529. https://doi.org/10.1002/mrd.22489.

(3) Wilkins, M. R.; Lindskog, I.; Gasteiger, E.; Bairoch, A.; Sanchez, J.-C.; Hochstrasser, D. F.; Appel, R. D. Detailed Peptide Characterization Using PEPTIDEMASS – a World-Wide-Web-Accessible Tool. *ELECTROPHORESIS* **1997**, *18* (3–4), 403–408. https://doi.org/10.1002/elps.1150180314.

(4) Virtanen, P.; Gommers, R.; Oliphant, T. E.; Haberland, M.; Reddy, T.; Cournapeau, D.; Burovski, E.; Peterson, P.; Weckesser, W.; Bright, J.; van der Walt, S. J.; Brett, M.; Wilson, J.; Millman, K. J.; Mayorov, N.; Nelson, A. R. J.; Jones, E.; Kern, R.; Larson, E.; Carey, C. J.; Polat, İ.; Feng, Y.; Moore, E. W.; VanderPlas, J.; Laxalde, D.; Perktold, J.; Cimrman, R.; Henriksen, I.; Quintero, E. A.; Harris, C. R.; Archibald, A. M.; Ribeiro, A. H.; Pedregosa, F.; van Mulbregt, P.; SciPy 1.0 Contributors. SciPy

1.0: Fundamental Algorithms for Scientific Computing in Python. *Nat Methods* **2020**, *17* (3), 261–272. https://doi.org/10.1038/s41592-019-0686-2.

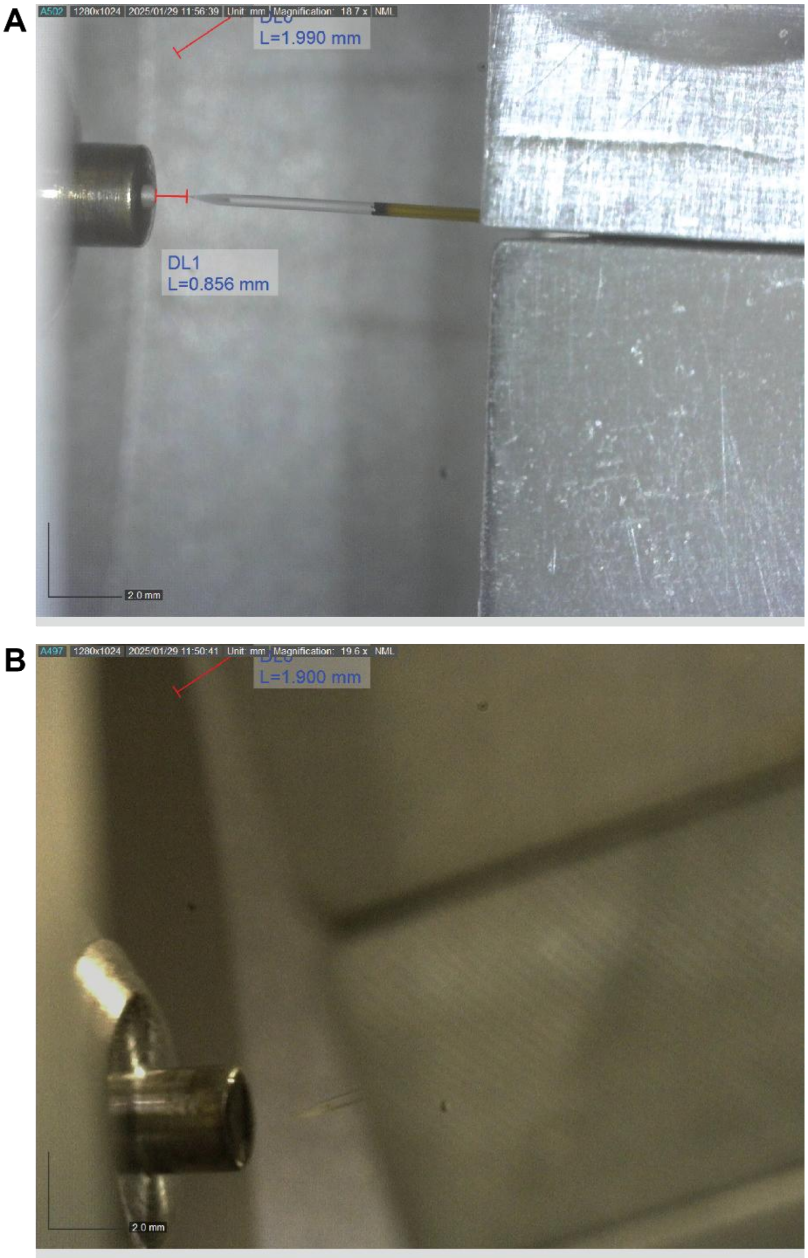

Figure S2: Emitter Position Images Recorded with Source Camera. (A) Top View of the emitter (x/z axes). The measured distance between the inlet capillary and the emitter tip was calibrated based on the known 360 μm O.D. of the silica capillary. Note that the 1.990 mm measurement is an artifact from the camera software. (B) Side view of the emitter (y/z axes). The angle of the emitter axis with respect to the inlet capillary axis was measured to be ~17.4° in ImageJ. Note that the 1.900 mm measurement is an artifact from the camera software.

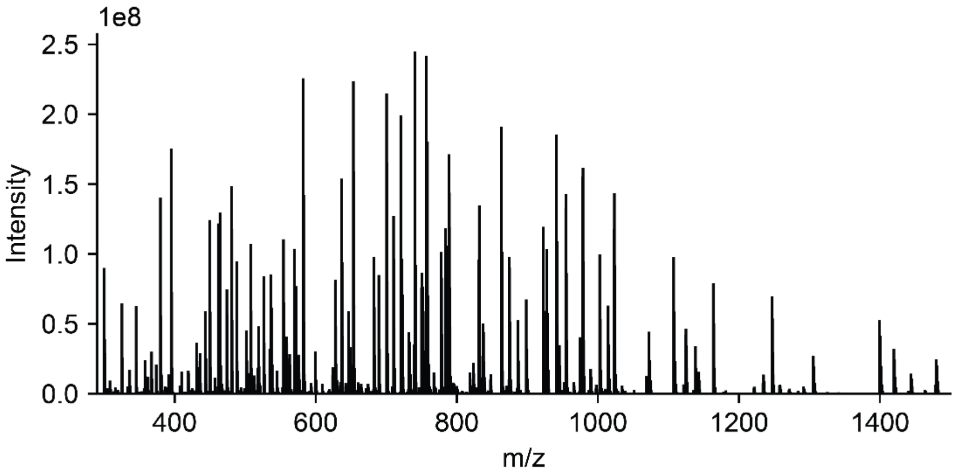

Figure S3: MS1 Spectra of Infused BSA Tryptic Digest. The Orbitrap MS1 spectra is shown for infusion of BSA peptides at x = 0 mm , y = 0 mm. z = -0.9 mm.

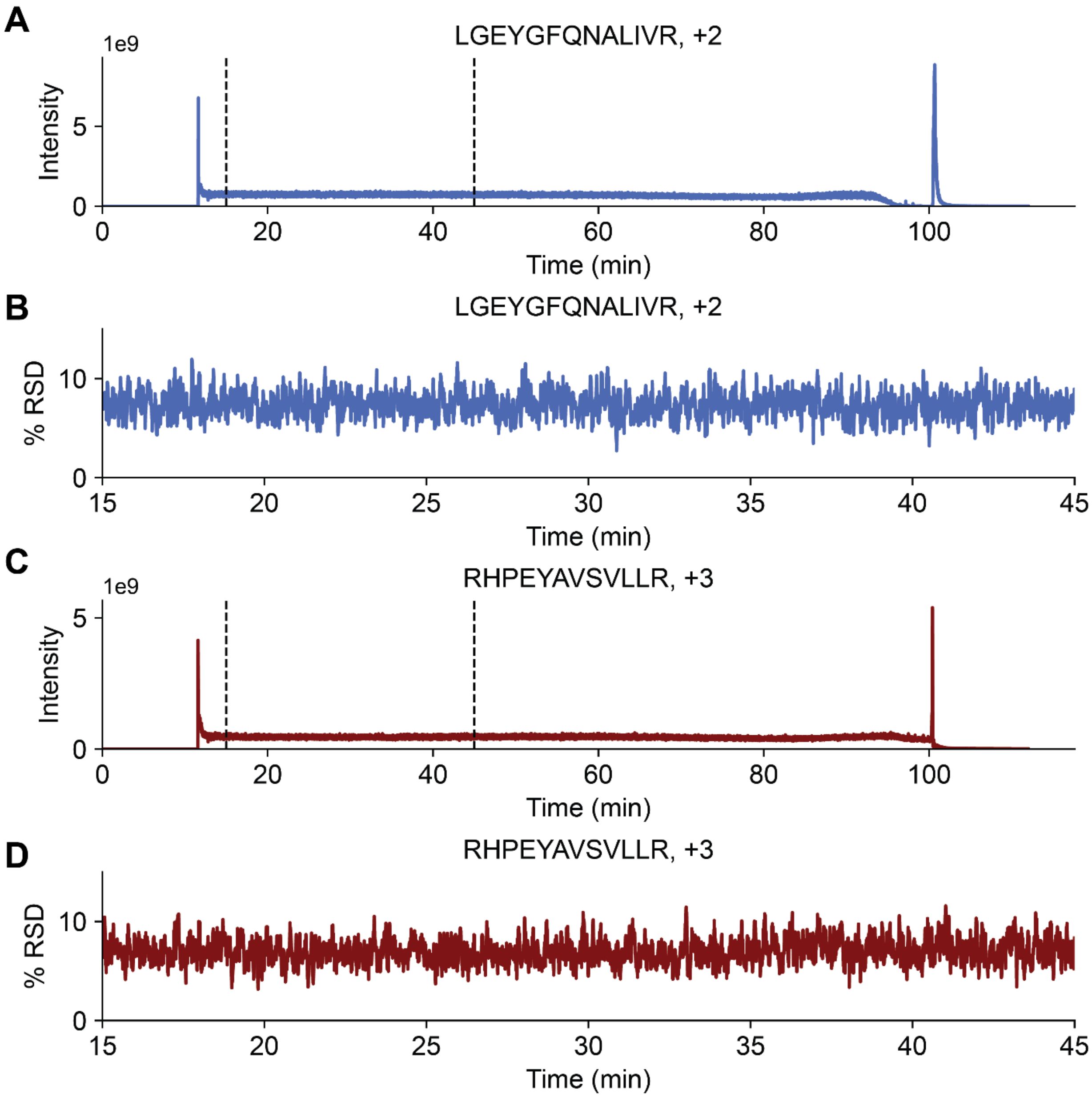

Figure S4: Signal Stability of Infused Peptides. (A) Signal stability over the infusion period for the $LGEYGFQNALIVR^{2+}$ ion. Dashed lines indicate the typical time window when emitter positioning data was collected during the sample infusion. (B) Plot showing the rolling %RSD value for the $LGEYGFQNALIVR^{2+}$ signal within the typical data collection window indicated in (A). (C) Signal stability over the infusion period for the $RHPEYAVSVLLR^{3+}$ ion. Dashed lines indicate the typical time window when emitter positioning data was collected during the sample infusion. (D) Plot showing the rolling %RSD value for the $RHPEYAVSVLLR^{3+\ +}$ signal within the typical data collection window indicated in (C). Rolling %RSD values were calculated over 20 scans.

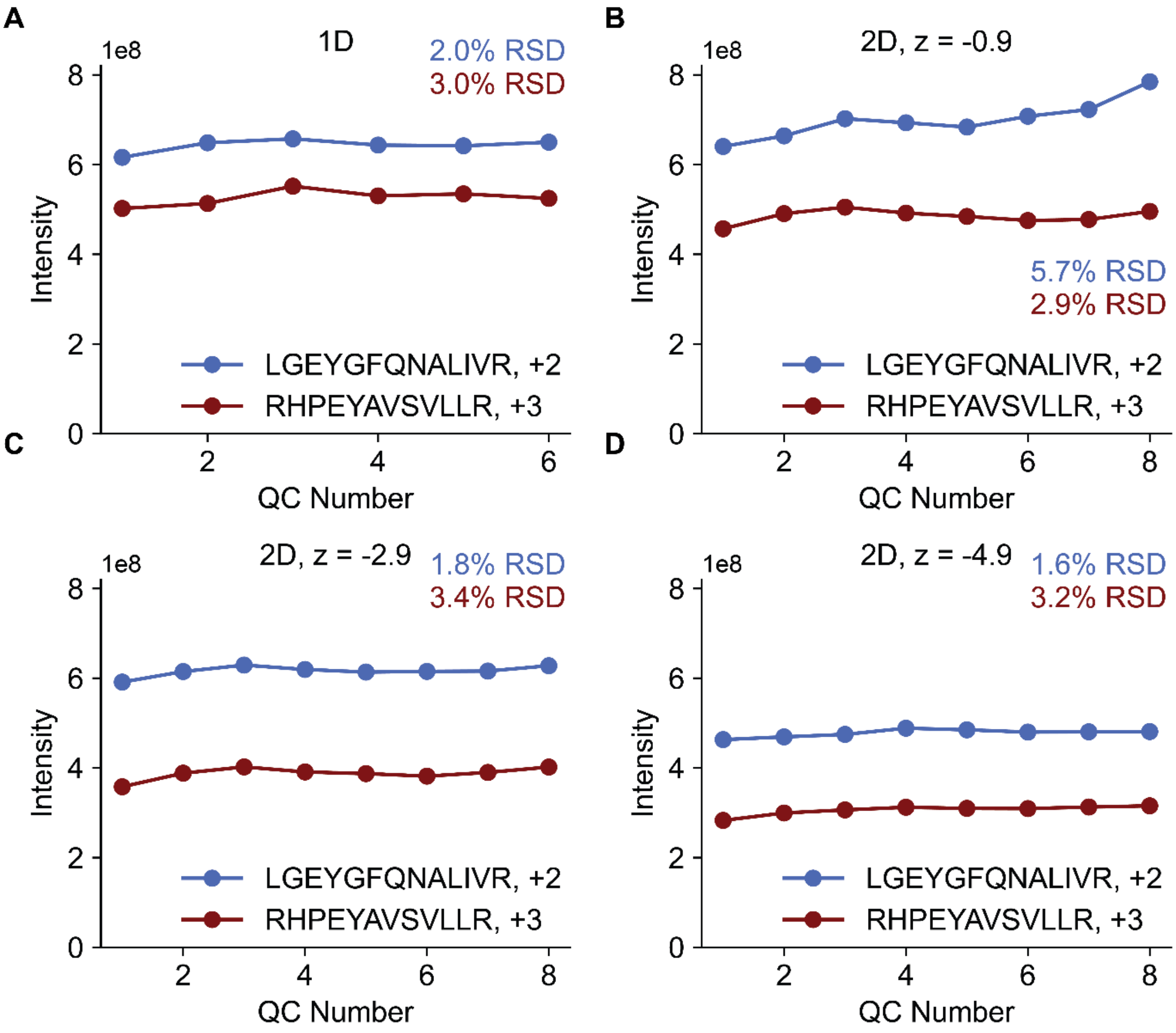

Figure S5. QC Measurements for Emitter Positioning Experiments. The position was returned to the starting position for each experiment periodically throughout data collection to confirm signal stability during the experiments. The signal intensity is shown for the (A) one-dimensional experiments shown in **Figure 1**, and the two-dimensional experimental shown in **Figure 2** and **Figure S10** at (B) z = -0.9, (C) z = -2.9, and (D) z = -4.9 mm. For all measurements, x = 0 mm and y = 0 mm. The relative standard deviations across QC measurements are shown on each plot with text in colors matching the corresponding peptide precursor data.

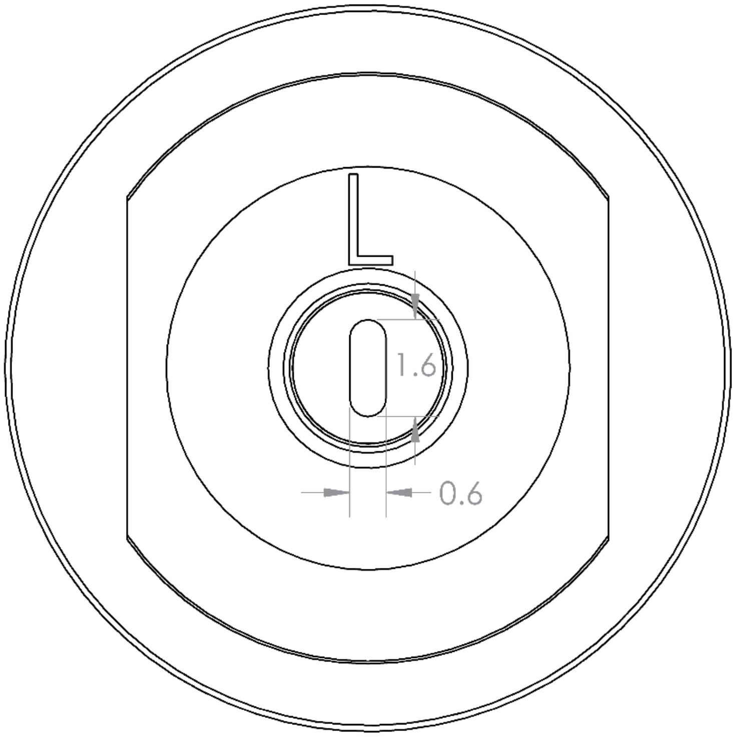

Figure S6. Inlet Capillary Drawing. A drawing of the high-capacity transfer tube (HCTT) is shown. Measurements are reported in mm with 0.6 mm being the width in the x dimension and 1.6 mm being the width in the y dimension.

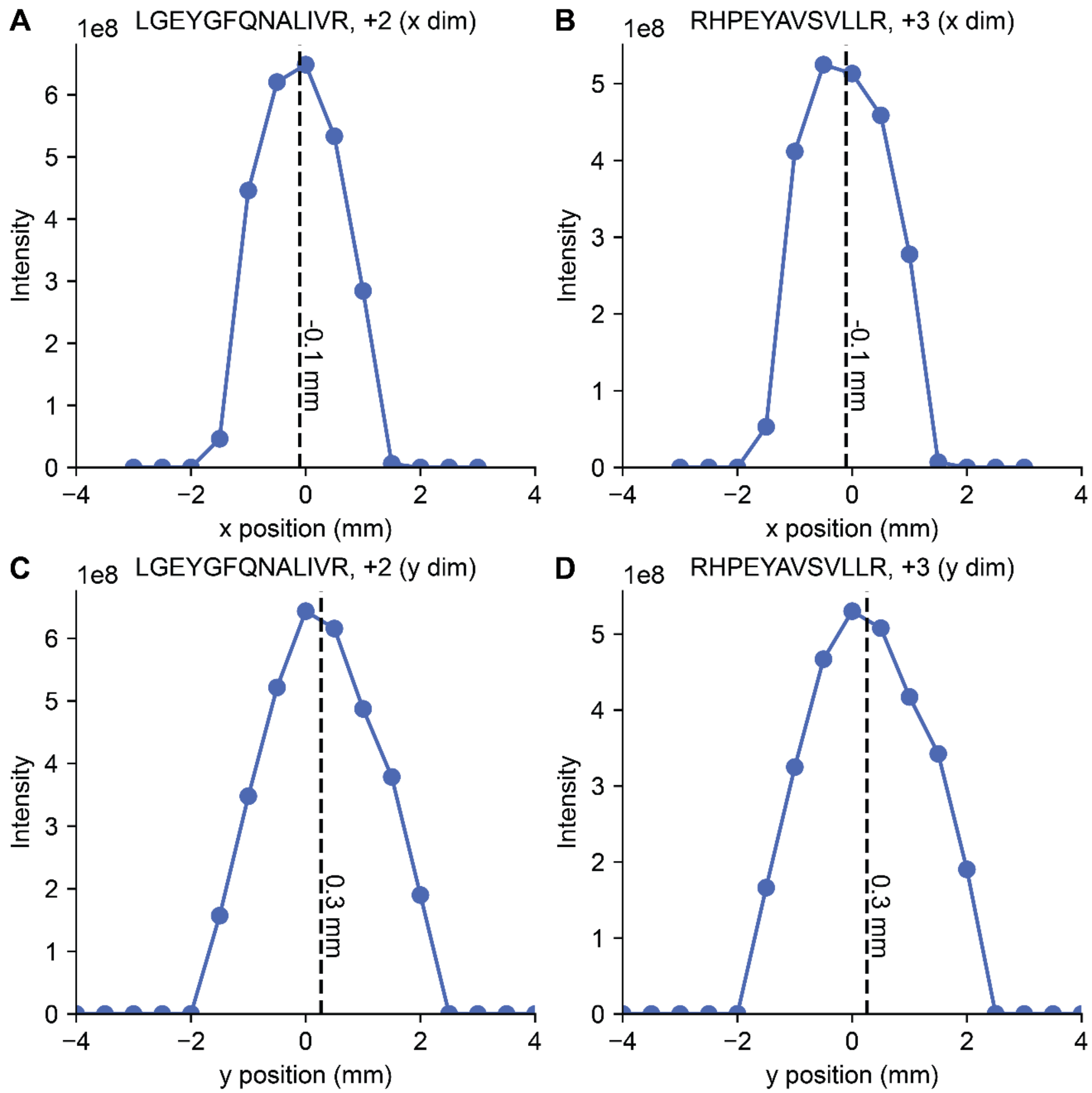

Figure S7. Estimating Distribution Centroids. For the data shown in **Figure 1C** and **1D**, the centroids of the distributions in the x- and y-dimensions (at z = -0.9) were determined. Data are shown for (A) LGEYGFQNALIVR (+2) for the x dimension, (B) RHPEYAVSVLLR (+3) for the x dimension, (C) LGEYGFQNALIVR (+2) for the y dimension, and (D) RHPEYAVSVLLR (+3) for the y dimension. Dotted black lines with text annotations indicate the position of the centroid calculated as $\bar{x} = \sum(x_i\, I_i)/\sum I_i$, where x_i is the x position for a datapoint and I_i is the corresponding intensity value at that position.

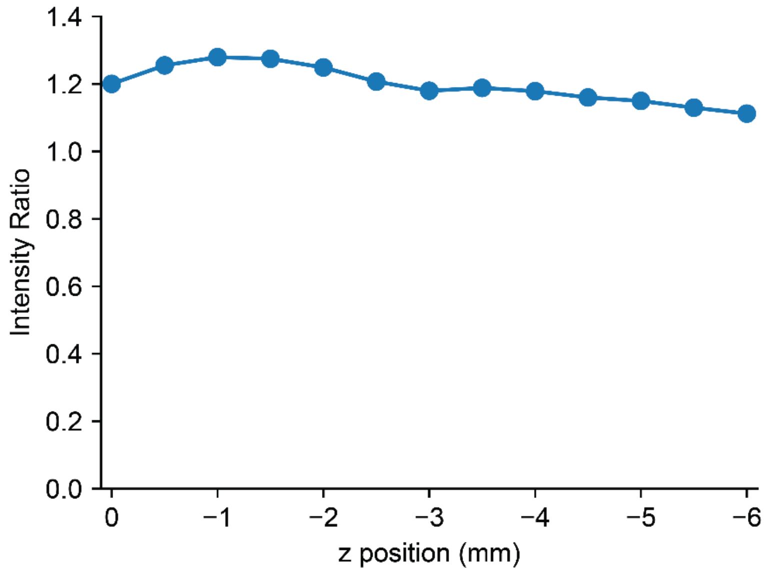

Figure S8. Dependence of Precursor Intensity Ratio on z Position The ratio of intensities for LGEYGFQNALIVR (+2) and RHPEYAVSVLLR (+3) is calculated and plotted as function of z position using the data shown in **Figure 1E** (at x = 0 and y = 0).

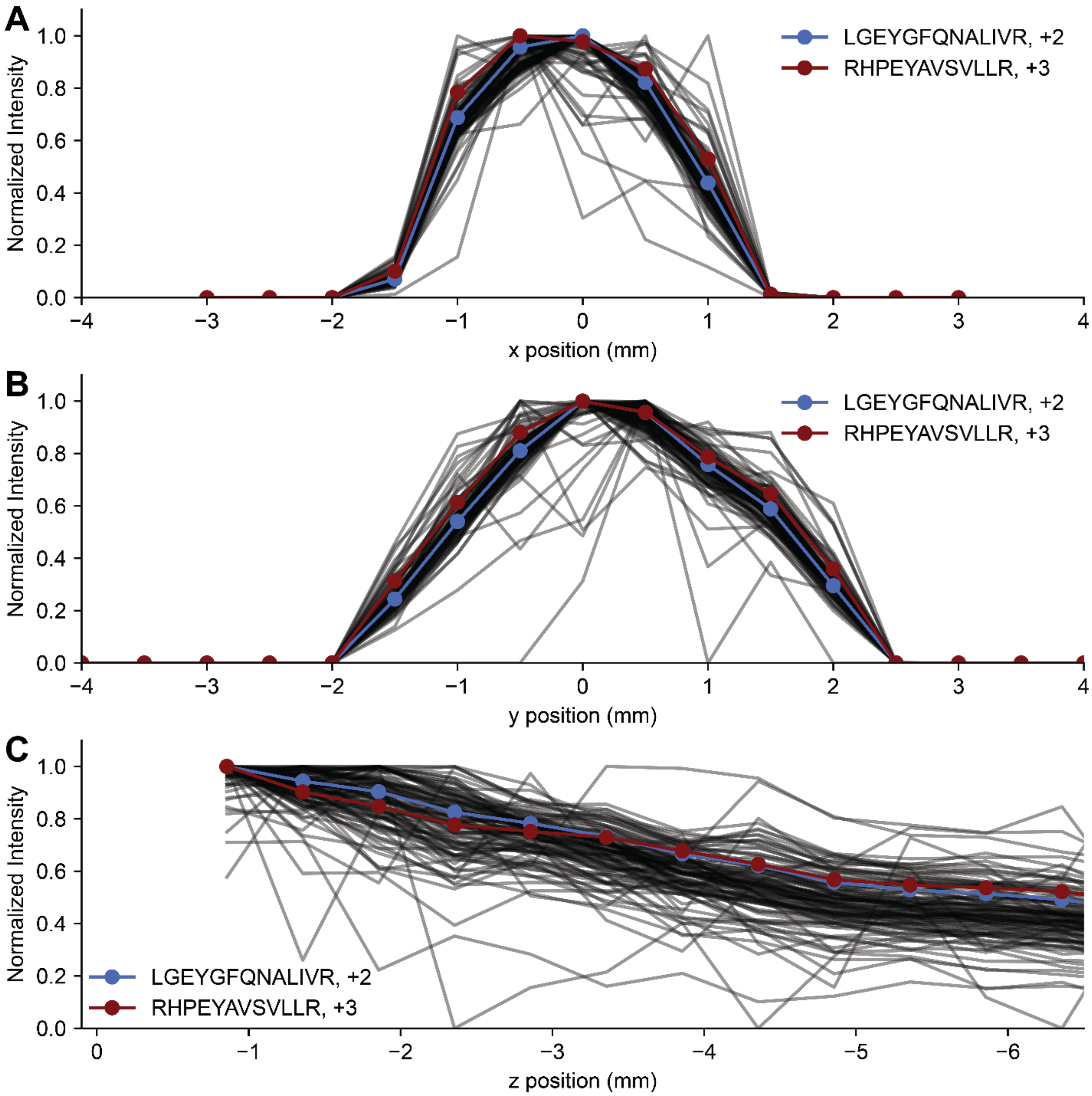

Figure S9. Dependence of Signal Intensity on Emitter Position for Low Level Precursor Ions. Mass spectra for the two extreme z-positions in **Figure 1E** (at x = 0 and y = 0) were filtered for m/z peaks present in both with S/N between 50 and 100 and charge >1 to represent lower-level intensity peaks. The normalized intensity dependence on position is shown for the (A) x dimension, (B), y dimension, and (C) z dimension. Normalized intensity profiles generated from the data shown in **Figure 1C-E** are overlaid to compare how these agree with the lower-level signals.

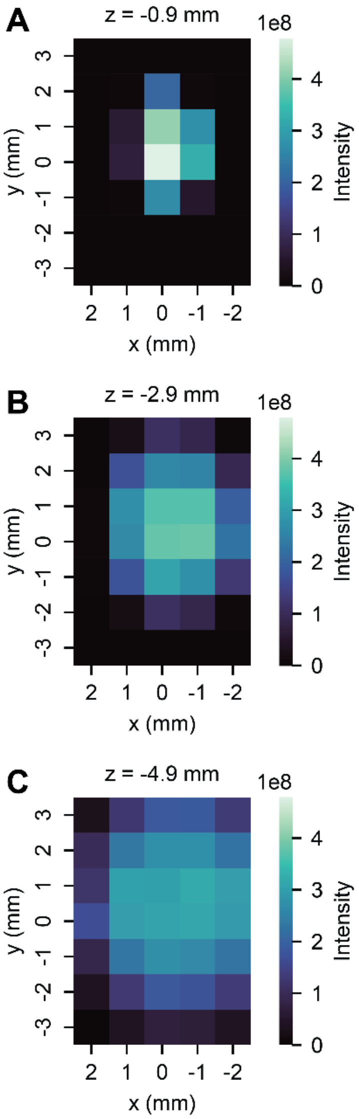

Figure S10. 2D Positioning Experiments for RHPEYAVSVLLR. Heatmaps showing the intensity of RHPEYAVSVLLR (+3) at different x/y positions at (A) z = -0.9 mm, (B) z = -2.9 mm, and (C) z = -4.9 mm.

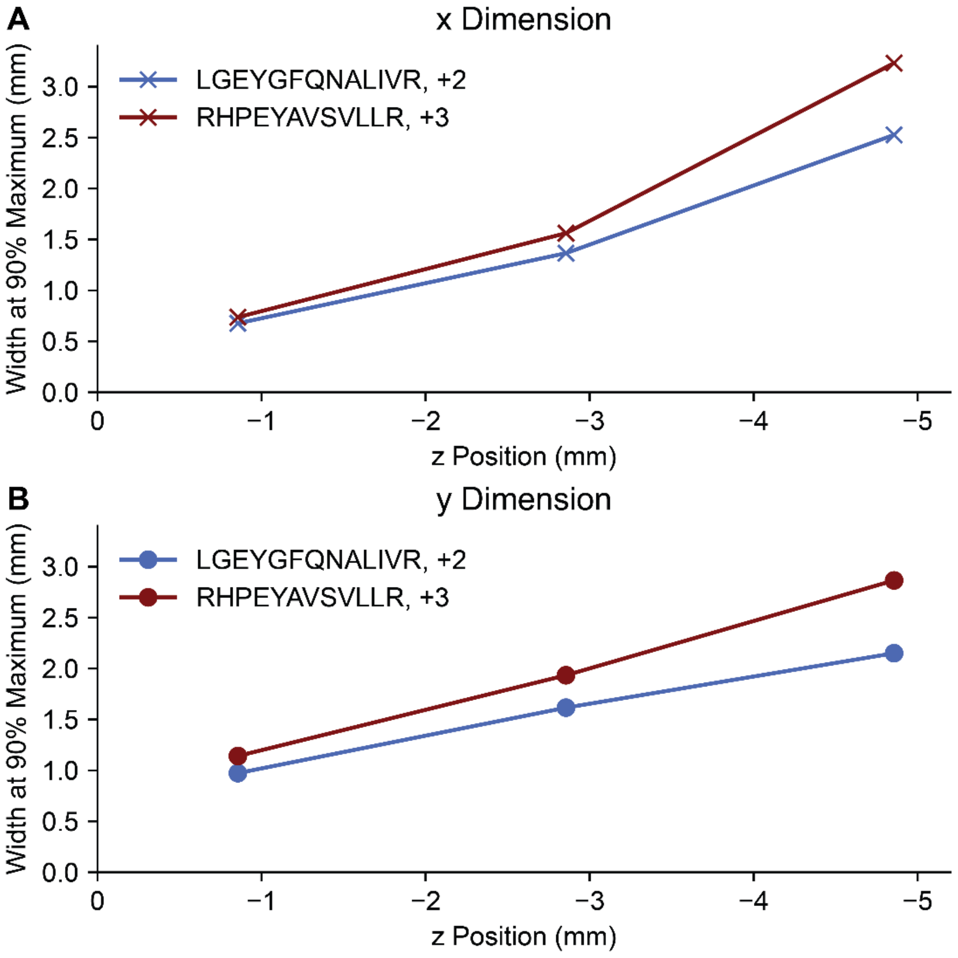

Figure S11. Width at 90% Maximum as Function of z Position. For the data shown in **Figure 2** and **S10**, the width at 90% maximum of the intensity distribution in the x- and y-dimensions were approximated by fitting the data to a spline interpolation function with SciPy. The approximate width values for the (A) x- and (B) y-distributions are then shown for the two different precursors ions as a function of the z position.

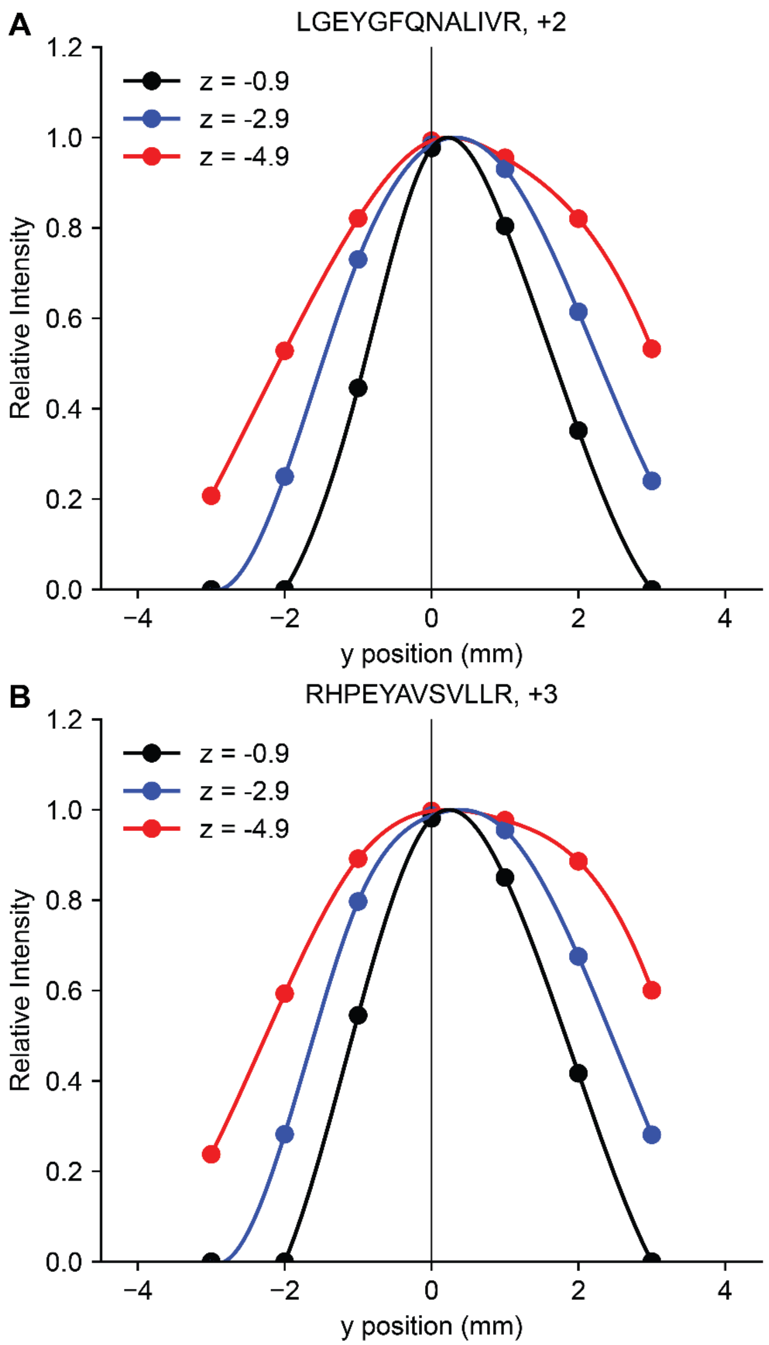

Figure S12. Dependence of y Intensity Distribution on z Position. For the data shown in **Figure 2** and **S10**, the y position-dependent normalized intensity distributions (at x = 0) were fit to a spline interpolation function with SciPy. Data are shown at different z positions for (A) LGEYGFQNALIVR (+2) and (B) RHPEYAVSVLLR (+3). The y = 0 point is indicated with a black line. Dots represent the experimental datapoints and the lines represent the spline function.

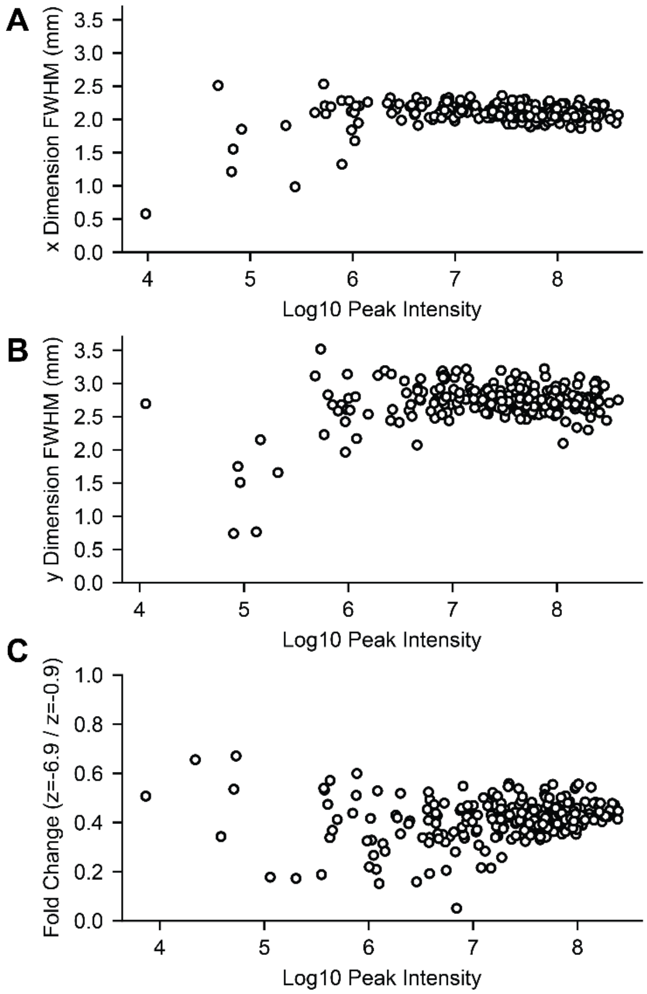

Figure S13. Dependence of Emitter Position Results on Peak Intensity. Mass spectra for the two extreme z-positions (at x = 0 and y = 0) were filtered for m/z peaks present in both spectra with S/N > 300 and charge > 1. The full-width half-maximum (FWHM) was approximated via spline interpolation for the (A) x-distribution (at z = -0.9 mm) and (B) y-distribution (at z = -0.9 mm), and (C) the fold change between the extreme z-positions (at x = 0, y = 0 mm) was calculated. These data show that low intensity peaks exhibit much greater variation.
